# Poly[bis­(μ-2-amino-4-nitro­benzoato)di-μ-aqua-dirubidium]

**DOI:** 10.1107/S1600536814008861

**Published:** 2014-04-30

**Authors:** Graham Smith

**Affiliations:** a Science and Engineering Faculty, Queensland University of Technology, GPO Box 2434, Brisbane, Queensland 4001, Australia

## Abstract

In the structure of the title salt, [Rb_2_(C_7_H_5_N_2_O_4_)_2_(H_2_O)_2_]_*n*_, the asymmetric unit comprises two independent and different seven-coordinate Rb^+^ cations, one forming an RbO_7_ polyhedron, the other a RbO_6_N polyhedron, each of which is considerably distorted. The RbO_7_ polyhedron comprises bridging O-atom donors from two water mol­ecules, three carboxyl­ate groups, and two nitro groups. The RbO_6_N polyhedron comprises the two bridging water mol­ecules, one monodentate amine N-atom donor, one carboxyl O-atom donor and three O-atom donors from nitro groups (one from the chelate bridge). The extension of the dinuclear unit gives a three-dimensional polymeric structure which is stabilized by both intra- and inter­molecular amine N—H⋯O and water O—H⋯O hydrogen bonds to carboxyl and water O-atom acceptors, as well as a number of inter-ring π–π inter­actions [minimum centroid–centroid separation = 3.364 (2) Å]. The title salt is isostructural with the analogous caesium salt.

## Related literature   

For the structures of some rubidium salts of substituted benzoic acids, see: Wiesbrock & Schmidbaur (2003 ▶); Dinnebier *et al.* (2002 ▶); Hu *et al.* (2005 ▶); Miao *et al.* (2011 ▶). For the structures of caesium 4-nitro­anthranilate and caesium 3,5-di­nitro­salicylate, see: Smith & Wermuth (2011 ▶) and Meng (2011 ▶), respectively. For the structures of the sodium and potassium 4-nitro­anthranilates, see: Smith (2013 ▶).
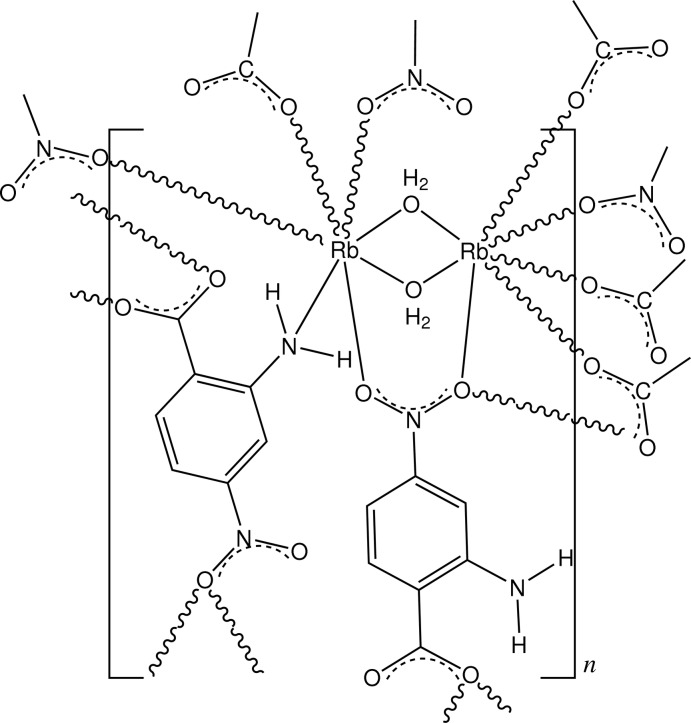


## Experimental   

### 

#### Crystal data  

[Rb_2_(C_7_H_5_N_2_O_4_)_2_(H_2_O)_2_]
*M* *_r_* = 569.23
Monoclinic, 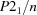

*a* = 15.2938 (9) Å
*b* = 6.8601 (3) Å
*c* = 17.8075 (10) Å
β = 99.996 (5)°
*V* = 1839.95 (17) Å^3^
*Z* = 4
Mo *K*α radiation
μ = 5.39 mm^−1^
*T* = 200 K
0.30 × 0.18 × 0.08 mm

#### Data collection  

Oxford Diffraction Gemini-S CCD diffractometer
Absorption correction: multi-scan (*CrysAlis PRO*; Agilent, 2013 ▶) *T*_min_ = 0.691, *T*_max_ = 0.980
6954 measured reflections
3634 independent reflections
2708 reflections with *I* > 2σ(*I*)
*R*_int_ = 0.046

#### Refinement  

*R*[*F* ^2^ > 2σ(*F* ^2^)] = 0.046
*wR*(*F* ^2^) = 0.075
*S* = 1.03
3634 reflections
295 parameters
8 restraints
H atoms treated by a mixture of independent and constrained refinement
Δρ_max_ = 0.61 e Å^−3^
Δρ_min_ = −0.51 e Å^−3^

### 

Data collection: *CrysAlis PRO* (Agilent, 2013 ▶); cell refinement: *CrysAlis PRO*; data reduction: *CrysAlis PRO*; program(s) used to solve structure: *SHELXS97* (Sheldrick, 2008 ▶); program(s) used to refine structure: *SHELXL97* (Sheldrick, 2008 ▶) within *WinGX* (Farrugia, 2012 ▶); molecular graphics: *PLATON* (Spek, 2009 ▶); software used to prepare material for publication: *PLATON*.

## Supplementary Material

Crystal structure: contains datablock(s) global, I. DOI: 10.1107/S1600536814008861/wm5020sup1.cif

Structure factors: contains datablock(s) I. DOI: 10.1107/S1600536814008861/wm5020Isup2.hkl

CCDC reference: 998206

Additional supporting information:  crystallographic information; 3D view; checkCIF report

## Figures and Tables

**Table 1:** Selected bond lengths (Å)

Rb1—O1*W*	3.041 (3)
Rb1—O2*W*	3.006 (3)
Rb1—O42*A*	3.064 (3)
Rb1—O42*A* ^i^	3.092 (3)
Rb1—O12*A* ^ii^	3.074 (3)
Rb1—O11*B* ^iii^	3.059 (3)
Rb1—O12*A* ^iv^	2.998 (3)
Rb2—O1*W*	2.994 (3)
Rb2—O2*W*	2.897 (3)
Rb2—O41*A*	2.992 (3)
Rb2—N2*B*	3.177 (4)
Rb2—O42*B* ^v^	2.984 (3)
Rb2—O12*B* ^vi^	2.947 (3)
Rb2—O42*B* ^iv^	3.069 (3)

Symmetry codes: (i) 
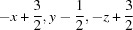
; (ii) 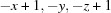; (iii) 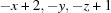; (iv) 
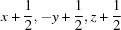
; (v) 
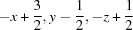
; (vi) 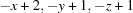.

**Table 2:** Hydrogen-bond geometry (Å, °)

*D*—H⋯*A*	*D*—H	H⋯*A*	*D*⋯*A*	*D*—H⋯*A*
N2*A*—H21*A*⋯O12*A*	0.91 (2)	1.97 (3)	2.686 (5)	134 (3)
N2*A*—H21*A*⋯O1*W* ^vii^	0.91 (2)	2.58 (4)	3.149 (5)	122 (3)
N2*A*—H22*A*⋯O11*B* ^v^	0.94 (3)	2.46 (3)	3.206 (5)	136 (3)
N2*B*—H21*B*⋯O11*A* ^viii^	0.90 (3)	2.01 (3)	2.831 (5)	151 (3)
N2*B*—H22*B*⋯O12*B*	0.90 (3)	1.88 (3)	2.644 (6)	142 (4)
O1*W*—H11*W*⋯O11*B* ^vi^	0.88 (3)	1.92 (4)	2.783 (4)	167 (4)
O1*W*—H12*W*⋯O12*A* ^viii^	0.89 (3)	1.96 (4)	2.847 (4)	176 (2)
O2*W*—H21*W*⋯O11*A* ^ii^	0.89 (4)	1.93 (4)	2.823 (4)	178 (7)
O2*W*—H22*W*⋯O12*B* ^iii^	0.88 (4)	1.95 (5)	2.812 (5)	166 (5)

Symmetry codes: (ii) 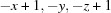; (iii) 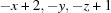; (v) 
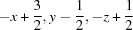
; (vi) 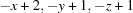; (vii) 
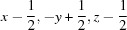
; (viii) 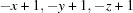.

**Fig. 1.**
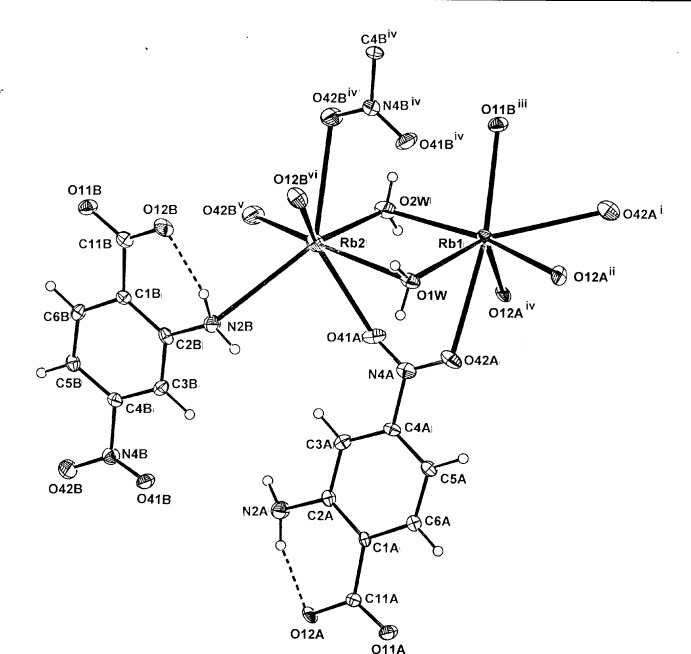
The molecular configuration and atom-numbering scheme for the dinuclear repeat unit of the title complex, with non-H atoms drawn as 30% probability displacement ellipsoids. Intramolecular hydrogen bonds are shown as dashed lines. For symmetry codes, see Table 1.

**Fig. 2.**
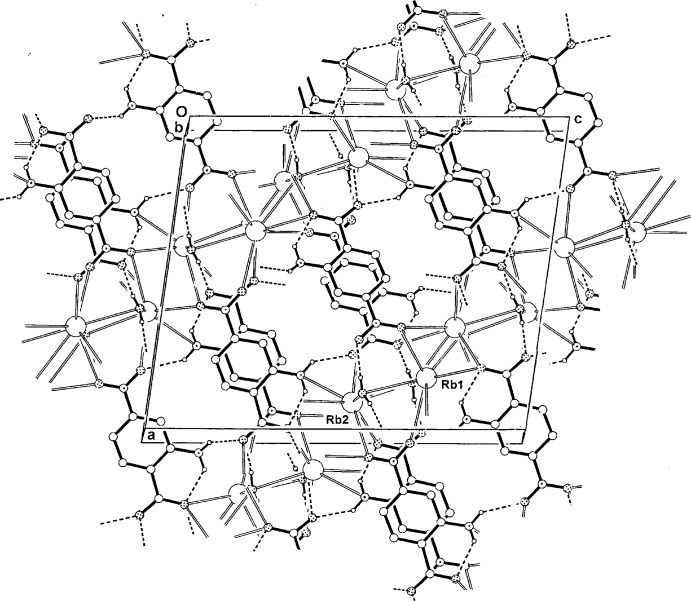
The polymeric structure in the unit cell viewed down b. Non-associative H atoms are omitted and hydrogen bonds are shown as dashed lines.

## supplementary crystallographic information

### 1. Comment

The structures of alkali metal salts derived from aromatic carboxylic acids are of interest (Smith, 2013), particularly with the heavier metals Rb and Cs, because of the expanded metals' coordination spheres and their ability to form coordination polymers. With 4-nitroanthranilic acid (4-NAA), a three-dimensional coordination polymeric structure [Cs_2_(C_7_H_5_N_2_O_4_)_2_(H_2_O)_2_] was described (Smith & Wermuth, 2011) and from the reaction of rubidium carbonate with 4-NAA, orange-red crystals of the title compound [Rb_2_(C_7_H_5_N_2_O_4_)_2_(H_2_O)_2_], were obtained, the structure of which is reported herein.

The Rb salt has the same formula as the Cs salt and has similar crystal data [comparative 200 K unit cell data for the Cs complex: *a* = 15.3615 (3), *b* = 6.9573 (2), *c* = 18.3714 (4) Å, β = 97.903 (2)°, *V* = 1944.79 (8) Å^3^, *Z* = 4, space group *P*2_1_/*n*]. The X-ray analysis reported here confirms that the Rb and Cs analogues are isotypic.

In the structure of the Rb salt, the dinuclear asymmetric unit contains two independent and different seven-coordinate Rb^+^ cations, with both having irregular coordination environments (Fig. 1). The RbO_7_ polyhedron about Rb1 comprises bridging oxygen donors from two water molecules, three carboxylate groups, and a nitro group, with one O atom doubly bridging [Rb—O range 2.998 (3)–3.092 (3) Å]. The RbO_6_N polyhedron about Rb2 comprises the two bridging water atoms, one monodentate amine N donor, one carboxyl O donor and three O donors from nitro groups (one doubly bridging) [Rb—O range 2.897 (3)–3.069 (3) Å] (Table 1). The Rb1···Rb2 separation in this dinuclear unit is 4.1208 (7) Å. Extension of this unit gives an overall three-dimensional polymeric structure (Fig. 2) which is stabilized by both intra- and intermolecular amine N—H···O and water O—H···O hydrogen bonds to both carboxyl and water O-atom acceptors (Table 2). Also, there are several inter-ring π···π interactions involving both ring 1 (C1*A*–C6*A*) and ring 2 (C1*B*–C6*B*) with a minimum ring centroid separation 1···1^viii^ of 3.364 (2) Å and a maximum ring centroid separation: 2···2^ix^ of 3.556 (2) Å [for symmetry code (viii), see Table 1; for symmetry code (ix) -*x* + 3/2, *y* + 1/2, -*z* + 1/2].

The minor difference between the two isotypic Rb and Cs salt structures is that in the description of the Cs salt, the coordination about Cs1 includes two longer Cs—O bonds to O41*B*^iv^ [3.326 (2) Å] (see Fig. 1) and to O1*W*1^i^ [3.414 (3) Å]. In the title Rb salt, the equivalent values [3.342 (3) and 3.495 (3) Å] preclude these as Rb—O bonds.

These structural features, including expanded metal coordination spheres and multiple bridging with polymeric extensions, are similar to those found in other Rb salts with substituted benzoic acids, *e.g.* rubidium 3,5-dinitrobenzoate (8-coordinate) (Miao *et al.*, 2011), rubidium anthranilate (7-coordinate) (Wiesbrock & Schmidbaur, 2003), rubidium salicylate (8-coordinate) (Dinnebier *et al.*, 2002) and rubidium 3,5-dinitosalicylate (10-coordinate) (Meng, 2011), this last Rb complex being isotypic with its Cs analogue (Hu *et al.*, 2005).

### 2. Experimental

The title compound was synthesized by heating together for 5 minutes, 0.1 mmol of rubidium carbonate and 0.2 mmol of 4-nitroanthranilic acid in 10 ml of 1:8 (*v*/*v*) ethanol–water. Partial room temperature evaporation of the solution gave orange-red flat prisms of the title complex from which a suitable specimen was cleaved for the X-ray analysis.

### 3. Refinement

The probability of isotypism with the Cs 4-nitroanthranilate monohydrate structure being recognized from the comparative cell data (Smith & Wermuth, 2011), the structure of the title complex was successfully phased in by inserting the non-H atoms from the Cs structure in the refinement. The same atom numbering scheme has been used for both structures. The amine and water H atoms were located in a difference-Fourier map and their positional and isotropic displacement parameters were allowed to ride with distance restraints on the N—H and O—H bond lengths of 0.91 (3)Å and with *U*_iso_(H) = 1.2*U*_eq_(N) or 1.5*U*_eq_(O). Other hydrogen atoms were included in the refinement in calculated positions with C—H = 0.95 Å and allowed to ride, with *U*_iso_(H) = 1.2*U*_eq_(C).

### Crystal data

**Table:**

[Rb_2_(C_7_H_5_N_2_O_4_)_2_(H_2_O)_2_]	*F*(000) = 1120
*M**_r_* = 569.23	*D*_x_ = 2.055 Mg m^−^^3^
Monoclinic, *P*2_1_/*n*	Mo *K*α radiation, λ = 0.71073 Å
Hall symbol: -P 2yn	Cell parameters from 1131 reflections
*a* = 15.2938 (9) Å	θ = 3.4–26.4°
*b* = 6.8601 (3) Å	µ = 5.39 mm^−^^1^
*c* = 17.8075 (10) Å	*T* = 200 K
β = 99.996 (5)°	Plate, orange red
*V* = 1839.95 (17) Å^3^	0.30 × 0.18 × 0.08 mm
*Z* = 4	

### Data collection

**Table:**

Oxford Diffraction Gemini-S CCD diffractometer	3634 independent reflections
Radiation source: Enhance (Mo) X-ray source	2708 reflections with *I* > 2σ(*I*)
Graphite monochromator	*R*_int_ = 0.046
Detector resolution: 16.077 pixels mm^-1^	θ_max_ = 26.0°, θ_min_ = 3.3°
ω scans	*h* = −15→18
Absorption correction: multi-scan (*CrysAlis PRO*; Agilent, 2013)	*k* = −7→8
*T*_min_ = 0.691, *T*_max_ = 0.980	*l* = −15→21
6954 measured reflections	

### Refinement

**Table:**

Refinement on *F*^2^	Primary atom site location: structure-invariant direct methods
Least-squares matrix: full	Secondary atom site location: difference Fourier map
*R*[*F*^2^ > 2σ(*F*^2^)] = 0.046	Hydrogen site location: inferred from neighbouring sites
*wR*(*F*^2^) = 0.075	H atoms treated by a mixture of independent and constrained refinement
*S* = 1.03	*w* = 1/[σ^2^(*F*_o_^2^) + (0.0163*P*)^2^] where *P* = (*F*_o_^2^ + 2*F*_c_^2^)/3
3634 reflections	(Δ/σ)_max_ = 0.001
295 parameters	Δρ_max_ = 0.61 e Å^−^^3^
8 restraints	Δρ_min_ = −0.51 e Å^−^^3^

### Special details

**Table:**

Geometry. Bond distances, angles *etc*. have been calculated using the rounded fractional coordinates. All su's are estimated from the variances of the (full) variance-covariance matrix. The cell e.s.d.'s are taken into account in the estimation of distances, angles and torsion angles
Refinement. Refinement of *F*^2^ against ALL reflections. The weighted *R*-factor *wR* and goodness of fit *S* are based on *F*^2^, conventional *R*-factors *R* are based on *F*, with *F* set to zero for negative *F*^2^. The threshold expression of *F*^2^ > σ(*F*^2^) is used only for calculating *R*-factors(gt) *etc*. and is not relevant to the choice of reflections for refinement. *R*-factors based on *F*^2^ are statistically about twice as large as those based on *F*, and *R*- factors based on ALL data will be even larger.

### Fractional atomic coordinates and isotropic or equivalent isotropic displacement parameters (Å^2^)

**Table:**

	*x*	*y*	*z*	*U*_iso_*/*U*_eq_	
Rb1	0.84018 (3)	−0.11232 (6)	0.71945 (2)	0.0291 (2)	
Rb2	0.90021 (3)	0.21443 (7)	0.54097 (3)	0.0333 (2)	
O1W	0.8411 (2)	0.3229 (4)	0.68713 (19)	0.0313 (11)	
O2W	0.8861 (2)	−0.2009 (5)	0.5656 (2)	0.0395 (12)	
O11A	0.26528 (19)	0.4400 (4)	0.47291 (17)	0.0316 (11)	
O11B	1.01609 (19)	0.4261 (4)	0.26964 (17)	0.0324 (11)	
O12A	0.29906 (19)	0.4056 (4)	0.35752 (16)	0.0312 (11)	
O12B	0.9810 (2)	0.4779 (5)	0.38434 (17)	0.0349 (11)	
O41A	0.7146 (2)	0.0750 (5)	0.5397 (2)	0.0404 (11)	
O41B	0.5388 (2)	0.4326 (5)	0.20204 (19)	0.0459 (13)	
O42A	0.6701 (2)	0.1017 (5)	0.64817 (19)	0.0394 (12)	
O42B	0.5826 (2)	0.4724 (5)	0.09497 (19)	0.0463 (14)	
N2A	0.4616 (3)	0.2771 (6)	0.3444 (2)	0.0345 (14)	
N2B	0.8117 (3)	0.4663 (6)	0.3981 (2)	0.0350 (14)	
N4A	0.6588 (2)	0.1193 (5)	0.5785 (2)	0.0293 (14)	
N4B	0.5972 (2)	0.4538 (5)	0.1643 (2)	0.0295 (12)	
C1A	0.4092 (3)	0.3222 (6)	0.4651 (2)	0.0174 (12)	
C1B	0.8639 (3)	0.4516 (6)	0.2769 (2)	0.0198 (12)	
C2A	0.4746 (3)	0.2689 (6)	0.4225 (2)	0.0219 (12)	
C2B	0.7949 (3)	0.4594 (6)	0.3196 (2)	0.0232 (14)	
C3A	0.5580 (3)	0.2062 (6)	0.4619 (2)	0.0234 (14)	
C3B	0.7072 (3)	0.4573 (6)	0.2809 (2)	0.0233 (14)	
C4A	0.5716 (3)	0.1917 (6)	0.5395 (2)	0.0206 (14)	
C4B	0.6909 (3)	0.4514 (6)	0.2033 (2)	0.0212 (14)	
C5A	0.5086 (3)	0.2394 (6)	0.5834 (3)	0.0233 (14)	
C5B	0.7571 (3)	0.4458 (6)	0.1594 (2)	0.0240 (14)	
C6A	0.4284 (3)	0.3055 (6)	0.5442 (2)	0.0206 (12)	
C6B	0.8427 (3)	0.4444 (6)	0.1980 (2)	0.0235 (14)	
C11A	0.3177 (3)	0.3931 (6)	0.4292 (3)	0.0213 (14)	
C11B	0.9617 (3)	0.4523 (6)	0.3130 (3)	0.0252 (16)	
H3A	0.60430	0.17440	0.43460	0.0280*	
H5A	0.51990	0.22740	0.63740	0.0280*	
H3B	0.65930	0.45980	0.30860	0.0280*	
H5B	0.74390	0.44300	0.10520	0.0290*	
H6A	0.38380	0.34160	0.57260	0.0250*	
H6B	0.88960	0.43830	0.16940	0.0280*	
H11W	0.882 (2)	0.414 (5)	0.695 (3)	0.0470*	
H12W	0.796 (2)	0.404 (5)	0.671 (3)	0.0470*	
H21A	0.4027 (14)	0.282 (6)	0.325 (2)	0.0420*	
H21B	0.771 (2)	0.503 (6)	0.426 (2)	0.0420*	
H21W	0.839 (2)	−0.278 (6)	0.553 (3)	0.0590*	
H22A	0.500 (2)	0.195 (5)	0.323 (2)	0.0420*	
H22B	0.8686 (15)	0.503 (6)	0.412 (3)	0.0420*	
H22W	0.927 (3)	−0.292 (6)	0.573 (3)	0.0590*	

### Atomic displacement parameters (Å^2^)

**Table:**

	*U*^11^	*U*^22^	*U*^33^	*U*^12^	*U*^13^	*U*^23^
Rb1	0.0343 (3)	0.0294 (3)	0.0220 (2)	0.0052 (2)	0.0004 (2)	−0.0010 (2)
Rb2	0.0369 (3)	0.0340 (3)	0.0255 (3)	−0.0029 (2)	−0.0039 (2)	−0.0018 (2)
O1W	0.0256 (18)	0.0285 (19)	0.036 (2)	−0.0001 (15)	−0.0055 (15)	0.0014 (16)
O2W	0.032 (2)	0.039 (2)	0.044 (2)	0.0029 (17)	−0.0035 (17)	0.0010 (19)
O11A	0.0209 (17)	0.050 (2)	0.0235 (18)	0.0042 (16)	0.0029 (14)	−0.0060 (16)
O11B	0.0211 (17)	0.042 (2)	0.033 (2)	−0.0003 (16)	0.0017 (14)	−0.0054 (17)
O12A	0.0328 (18)	0.045 (2)	0.0138 (17)	0.0093 (16)	−0.0012 (13)	0.0057 (15)
O12B	0.0358 (19)	0.045 (2)	0.0197 (18)	0.0043 (17)	−0.0065 (14)	−0.0050 (16)
O41A	0.0231 (18)	0.043 (2)	0.054 (2)	0.0041 (17)	0.0035 (17)	−0.0052 (19)
O41B	0.0234 (18)	0.074 (3)	0.042 (2)	−0.0015 (19)	0.0106 (16)	0.003 (2)
O42A	0.038 (2)	0.041 (2)	0.033 (2)	0.0045 (18)	−0.0113 (16)	0.0062 (17)
O42B	0.0323 (19)	0.075 (3)	0.029 (2)	−0.0023 (19)	−0.0023 (16)	0.0082 (19)
N2A	0.033 (2)	0.045 (3)	0.026 (2)	0.006 (2)	0.0067 (19)	−0.005 (2)
N2B	0.033 (2)	0.051 (3)	0.022 (2)	0.006 (2)	0.0075 (19)	0.000 (2)
N4A	0.025 (2)	0.018 (2)	0.042 (3)	−0.0049 (19)	−0.0024 (19)	0.001 (2)
N4B	0.028 (2)	0.031 (2)	0.029 (2)	−0.001 (2)	0.0032 (18)	−0.0013 (19)
C1A	0.023 (2)	0.013 (2)	0.016 (2)	−0.0031 (19)	0.0027 (18)	0.0016 (18)
C1B	0.023 (2)	0.015 (2)	0.021 (2)	0.003 (2)	0.0029 (19)	−0.0017 (19)
C2A	0.026 (2)	0.021 (2)	0.018 (2)	−0.003 (2)	0.0017 (19)	−0.001 (2)
C2B	0.034 (3)	0.015 (2)	0.020 (2)	0.003 (2)	0.003 (2)	0.0008 (19)
C3A	0.021 (2)	0.019 (2)	0.031 (3)	−0.003 (2)	0.007 (2)	−0.005 (2)
C3B	0.023 (2)	0.021 (2)	0.028 (3)	0.000 (2)	0.010 (2)	−0.001 (2)
C4A	0.017 (2)	0.014 (2)	0.028 (3)	−0.002 (2)	−0.0042 (19)	−0.001 (2)
C4B	0.020 (2)	0.014 (2)	0.028 (3)	−0.003 (2)	0.000 (2)	0.003 (2)
C5A	0.026 (2)	0.021 (3)	0.021 (2)	0.000 (2)	−0.0015 (19)	−0.001 (2)
C5B	0.028 (2)	0.025 (3)	0.018 (2)	−0.003 (2)	0.0014 (19)	−0.001 (2)
C6A	0.024 (2)	0.017 (2)	0.020 (2)	−0.003 (2)	0.0020 (19)	−0.0019 (19)
C6B	0.025 (2)	0.021 (2)	0.026 (3)	−0.004 (2)	0.009 (2)	−0.002 (2)
C11A	0.019 (2)	0.018 (2)	0.026 (3)	−0.002 (2)	0.0015 (19)	0.001 (2)
C11B	0.029 (3)	0.018 (2)	0.028 (3)	0.003 (2)	0.003 (2)	−0.001 (2)

### Geometric parameters (Å, º)

**Table:**

Rb1—O1W	3.041 (3)	N2B—C2B	1.378 (5)
Rb1—O2W	3.006 (3)	N4A—C4A	1.479 (5)
Rb1—O42A	3.064 (3)	N4B—C4B	1.480 (5)
Rb1—O42A^i^	3.092 (3)	N2A—H21A	0.91 (2)
Rb1—O12A^ii^	3.074 (3)	N2A—H22A	0.94 (3)
Rb1—O11B^iii^	3.059 (3)	N2B—H21B	0.90 (3)
Rb1—O12A^iv^	2.998 (3)	N2B—H22B	0.90 (3)
Rb2—O1W	2.994 (3)	C1A—C2A	1.405 (6)
Rb2—O2W	2.897 (3)	C1A—C6A	1.393 (5)
Rb2—O41A	2.992 (3)	C1A—C11A	1.514 (6)
Rb2—N2B	3.177 (4)	C1B—C2B	1.405 (6)
Rb2—O42B^v^	2.984 (3)	C1B—C6B	1.387 (5)
Rb2—O12B^vi^	2.947 (3)	C1B—C11B	1.522 (7)
Rb2—O42B^iv^	3.069 (3)	C2A—C3A	1.412 (6)
O11A—C11A	1.253 (6)	C2B—C3B	1.398 (6)
O11B—C11B	1.242 (6)	C3A—C4A	1.365 (5)
O12A—C11A	1.262 (6)	C3B—C4B	1.362 (5)
O12B—C11B	1.266 (6)	C4A—C5A	1.381 (6)
O41A—N4A	1.226 (5)	C4B—C5B	1.383 (6)
O41B—N4B	1.216 (5)	C5A—C6A	1.378 (6)
O42A—N4A	1.229 (5)	C5B—C6B	1.369 (6)
O42B—N4B	1.223 (5)	C3A—H3A	0.9500
O1W—H11W	0.88 (3)	C3B—H3B	0.9500
O1W—H12W	0.89 (3)	C5A—H5A	0.9500
O2W—H21W	0.89 (4)	C5B—H5B	0.9500
O2W—H22W	0.88 (4)	C6A—H6A	0.9500
N2A—C2A	1.372 (5)	C6B—H6B	0.9500
			
O1W—Rb1—O2W	90.95 (9)	Rb1—O2W—H22W	106 (3)
O1W—Rb1—O42A	58.84 (9)	H21W—O2W—H22W	98 (4)
O1W—Rb1—O42A^i^	140.33 (9)	Rb2—O2W—H22W	131 (3)
O1W—Rb1—O12A^ii^	125.72 (8)	Rb2—O2W—H21W	129 (3)
O1W—Rb1—O11B^iii^	132.54 (8)	Rb2—N2B—C2B	140.6 (3)
O1W—Rb1—O12A^iv^	72.50 (8)	O41A—N4A—C4A	118.6 (3)
O2W—Rb1—O42A	92.01 (9)	O41A—N4A—O42A	123.7 (3)
O2W—Rb1—O42A^i^	128.13 (9)	O42A—N4A—C4A	117.7 (3)
O2W—Rb1—O12A^ii^	73.42 (8)	O41B—N4B—O42B	123.2 (3)
O2W—Rb1—O11B^iii^	68.69 (9)	O41B—N4B—C4B	118.9 (3)
O2W—Rb1—O12A^iv^	163.43 (9)	O42B—N4B—C4B	117.9 (3)
O42A—Rb1—O42A^i^	117.91 (9)	C2A—N2A—H22A	113 (2)
O12A^ii^—Rb1—O42A	69.90 (8)	H21A—N2A—H22A	121 (3)
O11B^iii^—Rb1—O42A	155.88 (8)	C2A—N2A—H21A	110 (2)
O12A^iv^—Rb1—O42A	80.17 (8)	Rb2—N2B—H22B	71 (3)
O12A^ii^—Rb1—O42A^i^	78.56 (8)	C2B—N2B—H21B	123 (2)
O11B^iii^—Rb1—O42A^i^	68.66 (8)	Rb2—N2B—H21B	87 (2)
O12A^iv^—Rb1—O42A^i^	68.24 (8)	H21B—N2B—H22B	120 (4)
O11B^iii^—Rb1—O12A^ii^	90.13 (8)	C2B—N2B—H22B	107 (3)
O12A^ii^—Rb1—O12A^iv^	116.59 (8)	C6A—C1A—C11A	118.1 (4)
O11B^iii^—Rb1—O12A^iv^	122.11 (8)	C2A—C1A—C11A	123.2 (3)
O1W—Rb2—O2W	94.08 (9)	C2A—C1A—C6A	118.7 (4)
O1W—Rb2—O41A	69.92 (9)	C2B—C1B—C6B	119.0 (4)
O1W—Rb2—N2B	114.21 (10)	C2B—C1B—C11B	123.1 (3)
O1W—Rb2—O42B^v^	157.81 (9)	C6B—C1B—C11B	117.9 (4)
O1W—Rb2—O12B^vi^	71.65 (8)	N2A—C2A—C1A	123.1 (4)
O1W—Rb2—O42B^iv^	103.10 (9)	C1A—C2A—C3A	118.5 (3)
O2W—Rb2—O41A	65.87 (9)	N2A—C2A—C3A	118.4 (4)
O2W—Rb2—N2B	128.58 (10)	N2B—C2B—C3B	119.7 (4)
O2W—Rb2—O42B^v^	66.11 (10)	N2B—C2B—C1B	121.7 (4)
O2W—Rb2—O12B^vi^	133.82 (9)	C1B—C2B—C3B	118.7 (3)
O2W—Rb2—O42B^iv^	68.24 (9)	C2A—C3A—C4A	119.4 (4)
O41A—Rb2—N2B	84.03 (11)	C2B—C3B—C4B	119.5 (4)
O41A—Rb2—O42B^v^	91.89 (9)	C3A—C4A—C4A	123.9 (4)
O12B^vi^—Rb2—O41A	137.91 (9)	N4A—C4A—C5A	118.3 (3)
O41A—Rb2—O42B^iv^	132.73 (9)	N4A—C4A—C3A	117.8 (4)
O42B^v^—Rb2—N2B	74.83 (10)	C3B—C4B—C5B	123.5 (4)
O12B^vi^—Rb2—N2B	96.63 (10)	N4B—C4B—C5B	118.6 (3)
O42B^iv^—Rb2—N2B	135.71 (10)	N4B—C4B—C3B	117.9 (4)
O12B^vi^—Rb2—O42B^v^	129.07 (9)	C4A—C5A—C6A	116.0 (4)
O42B^v^—Rb2—O42B^iv^	79.55 (9)	C4B—C5B—C6B	116.5 (3)
O12B^vi^—Rb2—O42B^iv^	72.67 (9)	C1A—C6A—C5A	123.5 (4)
Rb1—O1W—Rb2	86.13 (8)	C1B—C6B—C5B	122.9 (4)
Rb1—O2W—Rb2	88.54 (9)	O12A—C11A—C1A	118.6 (4)
Rb1^iii^—O11B—C11B	127.9 (3)	O11A—C11A—O12A	123.7 (4)
Rb1^ii^—O12A—C11A	115.1 (3)	O11A—C11A—C1A	117.7 (4)
Rb1^vii^—O12A—C11A	145.0 (3)	O11B—C11B—O12B	125.4 (4)
Rb1^ii^—O12A—Rb1^vii^	99.88 (8)	O11B—C11B—C1B	116.9 (4)
Rb2^vi^—O12B—C11B	124.8 (3)	O12B—C11B—C1B	117.7 (4)
Rb2—O41A—N4A	132.0 (3)	C2A—C3A—H3A	120.00
Rb1—O42A—N4A	115.2 (2)	C4A—C3A—H3A	120.00
Rb1—O42A—Rb1^viii^	98.05 (9)	C2B—C3B—H3B	120.00
Rb1^viii^—O42A—N4A	133.7 (3)	C4B—C3B—H3B	120.00
Rb2^ix^—O42B—N4B	148.3 (3)	C4A—C5A—H5A	122.00
Rb2^vii^—O42B—N4B	105.6 (2)	C6A—C5A—H5A	122.00
Rb2^ix^—O42B—Rb2^vii^	100.45 (10)	C4B—C5B—H5B	122.00
Rb1—O1W—H12W	130 (2)	C6B—C5B—H5B	122.00
Rb2—O1W—H11W	90 (3)	C1A—C6A—H6A	118.00
Rb2—O1W—H12W	102 (3)	C5A—C6A—H6A	118.00
H11W—O1W—H12W	96 (3)	C1B—C6B—H6B	119.00
Rb1—O1W—H11W	134 (2)	C5B—C6B—H6B	118.00
Rb1—O2W—H21W	93 (3)		
			
O2W—Rb1—O1W—Rb2	−4.05 (8)	O1W—Rb2—O12B^vi^—C11B^vi^	30.1 (3)
O42A—Rb1—O1W—Rb2	−95.84 (10)	O2W—Rb2—O12B^vi^—C11B^vi^	−47.5 (4)
O42A^i^—Rb1—O1W—Rb2	166.82 (10)	O41A—Rb2—O12B^vi^—C11B^vi^	55.0 (4)
O12A^ii^—Rb1—O1W—Rb2	−74.20 (10)	N2B—Rb2—O12B^vi^—C11B^vi^	143.4 (3)
O11B^iii^—Rb1—O1W—Rb2	57.38 (13)	O1W—Rb2—O42B^iv^—Rb2^iii^	157.48 (9)
O12A^iv^—Rb1—O1W—Rb2	175.23 (10)	O1W—Rb2—O42B^iv^—N4B^iv^	−4.3 (3)
O1W—Rb1—O2W—Rb2	4.18 (9)	O2W—Rb2—O42B^iv^—Rb2^iii^	68.29 (10)
O42A—Rb1—O2W—Rb2	63.04 (9)	O2W—Rb2—O42B^iv^—N4B^iv^	−93.5 (3)
O42A^i^—Rb1—O2W—Rb2	−168.43 (8)	O41A—Rb2—O42B^iv^—Rb2^iii^	82.84 (14)
O12A^ii^—Rb1—O2W—Rb2	131.36 (9)	O41A—Rb2—O42B^iv^—N4B^iv^	−79.0 (3)
O11B^iii^—Rb1—O2W—Rb2	−131.82 (10)	N2B—Rb2—O42B^iv^—Rb2^iii^	−55.24 (16)
O1W—Rb1—O42A—N4A	85.4 (3)	N2B—Rb2—O42B^iv^—N4B^iv^	142.9 (2)
O1W—Rb1—O42A—Rb1^viii^	−61.55 (10)	Rb1^iii^—O11B—C11B—O12B	55.8 (6)
O2W—Rb1—O42A—N4A	−4.5 (3)	Rb1^iii^—O11B—C11B—C1B	−123.6 (3)
O2W—Rb1—O42A—Rb1^viii^	−151.44 (9)	Rb1^ii^—O12A—C11A—O11A	−69.9 (5)
O42A^i^—Rb1—O42A—N4A	−140.4 (3)	Rb1^ii^—O12A—C11A—C1A	111.6 (3)
O42A^i^—Rb1—O42A—Rb1^viii^	72.69 (11)	Rb1^vii^—O12A—C11A—O11A	112.4 (5)
O12A^ii^—Rb1—O42A—N4A	−76.1 (3)	Rb1^vii^—O12A—C11A—C1A	−66.1 (6)
O12A^ii^—Rb1—O42A—Rb1^viii^	137.04 (10)	Rb2^vi^—O12B—C11B—O11B	56.1 (5)
O11B^iii^—Rb1—O42A—N4A	−40.3 (4)	Rb2^vi^—O12B—C11B—C1B	−124.4 (3)
O11B^iii^—Rb1—O42A—Rb1^viii^	172.79 (15)	Rb2—O41A—N4A—O42A	−59.4 (5)
O12A^iv^—Rb1—O42A—N4A	160.8 (3)	Rb2—O41A—N4A—C4A	122.8 (3)
O12A^iv^—Rb1—O42A—Rb1^viii^	13.87 (8)	Rb1—O42A—N4A—O41A	−7.6 (5)
O1W—Rb1—O42A^i^—Rb1^i^	119.97 (12)	Rb1—O42A—N4A—C4A	170.2 (3)
O1W—Rb1—O42A^i^—N4A^i^	−16.9 (4)	Rb1^viii^—O42A—N4A—O41A	124.0 (4)
O2W—Rb1—O42A^i^—Rb1^i^	−71.66 (12)	Rb1^viii^—O42A—N4A—C4A	−58.2 (5)
O2W—Rb1—O42A^i^—N4A^i^	151.4 (3)	Rb2^ix^—O42B—N4B—O41B	120.8 (5)
O42A—Rb1—O42A^i^—Rb1^i^	46.14 (12)	Rb2^ix^—O42B—N4B—C4B	−61.0 (6)
O42A—Rb1—O42A^i^—N4A^i^	−90.8 (3)	Rb2^vii^—O42B—N4B—O41B	−23.4 (4)
O1W—Rb1—O12A^ii^—Rb1^i^	−131.30 (9)	Rb2^vii^—O42B—N4B—C4B	154.7 (3)
O1W—Rb1—O12A^ii^—C11A^ii^	47.4 (3)	Rb2—N2B—C2B—C1B	−62.6 (6)
O2W—Rb1—O12A^ii^—Rb1^i^	149.82 (10)	Rb2—N2B—C2B—C3B	116.5 (5)
O2W—Rb1—O12A^ii^—C11A^ii^	−31.5 (3)	O41A—N4A—C4A—C3A	−0.3 (6)
O42A—Rb1—O12A^ii^—Rb1^i^	−111.67 (10)	O41A—N4A—C4A—C5A	179.3 (4)
O42A—Rb1—O12A^ii^—C11A^ii^	67.0 (3)	O42A—N4A—C4A—C3A	−178.2 (4)
O1W—Rb1—O11B^iii^—C11B^iii^	−44.3 (4)	O42A—N4A—C4A—C5A	1.4 (6)
O2W—Rb1—O11B^iii^—C11B^iii^	26.2 (3)	O41B—N4B—C4B—C3B	−10.5 (6)
O42A—Rb1—O11B^iii^—C11B^iii^	65.0 (4)	O41B—N4B—C4B—C5B	170.3 (4)
O1W—Rb1—O12A^iv^—Rb1^viii^	46.25 (8)	O42B—N4B—C4B—C3B	171.3 (4)
O1W—Rb1—O12A^iv^—C11A^iv^	−131.6 (5)	O42B—N4B—C4B—C5B	−7.9 (6)
O42A—Rb1—O12A^iv^—Rb1^viii^	−14.02 (8)	C6A—C1A—C2A—N2A	178.9 (4)
O42A—Rb1—O12A^iv^—C11A^iv^	168.1 (5)	C6A—C1A—C2A—C3A	−1.9 (6)
O2W—Rb2—O1W—Rb1	4.22 (9)	C11A—C1A—C2A—N2A	0.3 (7)
O41A—Rb2—O1W—Rb1	66.67 (9)	C11A—C1A—C2A—C3A	179.5 (4)
N2B—Rb2—O1W—Rb1	140.07 (10)	C2A—C1A—C6A—C5A	0.1 (6)
O42B^v^—Rb2—O1W—Rb1	30.1 (3)	C11A—C1A—C6A—C5A	178.8 (4)
O12B^vi^—Rb2—O1W—Rb1	−130.83 (10)	C2A—C1A—C11A—O11A	−178.7 (4)
O42B^iv^—Rb2—O1W—Rb1	−64.38 (9)	C2A—C1A—C11A—O12A	−0.2 (6)
O1W—Rb2—O2W—Rb1	−4.26 (9)	C6A—C1A—C11A—O11A	2.7 (6)
O41A—Rb2—O2W—Rb1	−70.11 (9)	C6A—C1A—C11A—O12A	−178.8 (4)
N2B—Rb2—O2W—Rb1	−129.90 (12)	C6B—C1B—C2B—N2B	179.8 (4)
O42B^v^—Rb2—O2W—Rb1	−173.89 (11)	C6B—C1B—C2B—C3B	0.7 (6)
O12B^vi^—Rb2—O2W—Rb1	64.09 (13)	C11B—C1B—C2B—N2B	−0.7 (6)
O42B^iv^—Rb2—O2W—Rb1	98.23 (10)	C11B—C1B—C2B—C3B	−179.8 (4)
O1W—Rb2—O41A—N4A	17.2 (3)	C2B—C1B—C6B—C5B	0.4 (6)
O2W—Rb2—O41A—N4A	121.5 (4)	C11B—C1B—C6B—C5B	−179.1 (4)
N2B—Rb2—O41A—N4A	−101.3 (4)	C2B—C1B—C11B—O11B	173.3 (4)
O42B^v^—Rb2—O41A—N4A	−175.8 (4)	C2B—C1B—C11B—O12B	−6.2 (6)
O12B^vi^—Rb2—O41A—N4A	−8.0 (4)	C6B—C1B—C11B—O11B	−7.2 (6)
O42B^iv^—Rb2—O41A—N4A	106.7 (4)	C6B—C1B—C11B—O12B	173.3 (4)
O1W—Rb2—N2B—C2B	−159.6 (5)	N2A—C2A—C3A—C4A	−178.2 (4)
O2W—Rb2—N2B—C2B	−42.3 (5)	C1A—C2A—C3A—C4A	2.6 (6)
O41A—Rb2—N2B—C2B	−94.7 (5)	N2B—C2B—C3B—C4B	179.7 (4)
O42B^v^—Rb2—N2B—C2B	−1.1 (5)	C1B—C2B—C3B—C4B	−1.2 (6)
O12B^vi^—Rb2—N2B—C2B	127.6 (5)	C2A—C3A—C4A—N4A	178.0 (4)
O42B^iv^—Rb2—N2B—C2B	55.7 (5)	C2A—C3A—C4A—C5A	−1.6 (7)
O1W—Rb2—O42B^v^—N4B^v^	115.8 (5)	C2B—C3B—C4B—N4B	−178.7 (4)
O1W—Rb2—O42B^v^—Rb2^iii^	−99.1 (2)	C2B—C3B—C4B—C5B	0.5 (6)
O2W—Rb2—O42B^v^—N4B^v^	144.2 (5)	N4A—C4A—C5A—C6A	−179.8 (4)
O2W—Rb2—O42B^v^—Rb2^iii^	−70.70 (10)	C3A—C4A—C5A—C6A	−0.2 (6)
O41A—Rb2—O42B^v^—N4B^v^	81.7 (5)	N4B—C4B—C5B—C6B	179.8 (4)
O41A—Rb2—O42B^v^—Rb2^iii^	−133.17 (10)	C3B—C4B—C5B—C6B	0.7 (6)
N2B—Rb2—O42B^v^—N4B^v^	−1.6 (5)	C4A—C5A—C6A—C1A	1.0 (6)
N2B—Rb2—O42B^v^—Rb2^iii^	143.53 (12)	C4B—C5B—C6B—C1B	−1.1 (6)

Symmetry codes: (i) −*x*+3/2, *y*−1/2, −*z*+3/2; (ii) −*x*+1, −*y*, −*z*+1; (iii) −*x*+2, −*y*, −*z*+1; (iv) *x*+1/2, −*y*+1/2, *z*+1/2; (v) −*x*+3/2, *y*−1/2, −*z*+1/2; (vi) −*x*+2, −*y*+1, −*z*+1; (vii) *x*−1/2, −*y*+1/2, *z*−1/2; (viii) −*x*+3/2, *y*+1/2, −*z*+3/2; (ix) −*x*+3/2, *y*+1/2, −*z*+1/2.

### Hydrogen-bond geometry (Å, º)

**Table:**

*D*—H···*A*	*D*—H	H···*A*	*D*···*A*	*D*—H···*A*
N2*A*—H21*A*···O12*A*	0.91 (2)	1.97 (3)	2.686 (5)	134 (3)
N2*A*—H21*A*···O1*W*^vii^	0.91 (2)	2.58 (4)	3.149 (5)	122 (3)
N2*A*—H22*A*···O11*B*^v^	0.94 (3)	2.46 (3)	3.206 (5)	136 (3)
N2*B*—H21*B*···O11*A*^x^	0.90 (3)	2.01 (3)	2.831 (5)	151 (3)
N2*B*—H22*B*···O12*B*	0.90 (3)	1.88 (3)	2.644 (6)	142 (4)
O1*W*—H11*W*···O11*B*^vi^	0.88 (3)	1.92 (4)	2.783 (4)	167 (4)
O1*W*—H12*W*···O12*A*^x^	0.89 (3)	1.96 (4)	2.847 (4)	176 (2)
O2*W*—H21*W*···O11*A*^ii^	0.89 (4)	1.93 (4)	2.823 (4)	178 (7)
O2*W*—H22*W*···O12*B*^iii^	0.88 (4)	1.95 (5)	2.812 (5)	166 (5)
C5*A*—H5*A*···O11*B*^xi^	0.95	2.59	3.488 (6)	158
C6*A*—H6*A*···O11*A*	0.95	2.40	2.755 (5)	101
C6*B*—H6*B*···O11*B*	0.95	2.40	2.739 (5)	101

Symmetry codes: (ii) −*x*+1, −*y*, −*z*+1; (iii) −*x*+2, −*y*, −*z*+1; (v) −*x*+3/2, *y*−1/2, −*z*+1/2; (vi) −*x*+2, −*y*+1, −*z*+1; (vii) *x*−1/2, −*y*+1/2, *z*−1/2; (x) −*x*+1, −*y*+1, −*z*+1; (xi) *x*−1/2, −*y*+1/2, *z*+1/2.
